# Positive Effects of Plyometric vs. Eccentric-Overload Training on Performance in Young Male Handball Players

**DOI:** 10.3390/jfmk8030113

**Published:** 2023-08-08

**Authors:** Eduardo Saez de Villareal, Julio Calleja-González, Pedro E. Alcaraz, Javier Feito-Blanco, Rodrígo Ramírez-Campillo

**Affiliations:** 1 Physical Performance Sports Research Center (PPSRC), Universidad Pablo Olavide Sevilla, 41013 Sevilla, Spain; eddysaezdevillarreal@gmail.com (E.S.d.V.);; 2 Physical Education and Sport Department, Faculty of Education and Sport, University of the Basque Country (UPV/EHU), 01007 Vitoria, Spain; 3 Research Center for High Performance Sport, Faculty of Sport Sciences, Catholic University of Murcia, 30107 Murcia, Spain; 4 Exercise and Rehabilitation Sciences Institute, School of Physical Therapy, Faculty of Rehabilitation Sciences, Universidad Andres Bello, Santiago 8370146, Chile

**Keywords:** plyometric exercise, musculoskeletal and neural physiological phenomena, human physical conditioning, team sports, movement, resistance training

## Abstract

This study aimed to compare the effects of two 8-week in-season strength-training programs on handball players’ physical and technical parameters. Thirty-six male athletes were randomly separated into three groups: a control group (*n* = 12), a plyometric training group (PG, *n* = 12), and an eccentric-overload training group (EG, *n* = 12). The PG and EG performed upper- and lower-limb plyometric or eccentric-overload exercises, respectively, three times per week. Control groups performed regular handball training. The athletes were assessed for counter movement jump (CMJ) and Abalakov vertical jump (ABK) height, 15 m linear sprint time, handball-throwing speed (i.e., penalty throw; 3-step running throw; jump throw), and cardiorespiratory endurance through the 20 m shuttle-run test. Heart rate and blood lactate were measured at the end of the endurance test. No baseline differences were noted for dependent variables between groups. The session rating of perceived exertion was similar between the intervention groups (PG = 361 ± 12.2 AU; EG = 370 ± 13.3 AU). The ANOVA revealed significant (*p* < 0.05; Δ = 5–9%; effect size (ES) = 0.45–1.96). Similar improvements for experimental groups compared to the control group for CMJ, ABK jump, penalty throw, 3-step running throw, and jump throw. However, interventions did not affect 15 m, cardiorespiratory endurance, nor heart rate or blood lactate after the endurance test. In conclusion, an 8-week handball intervention by performing plyometric or eccentric-overload training in-season improves the physical and technical parameters of male players when compared to regular handball practice.

## 1. Introduction

Handball is a high-intensity intermittent sport, demanding a player’s optimal levels of endurance and strength, and a predominance of technical parameters, such as passing, catching, throwing, checking, and blocking [1]. It additionally involves movements including running, jumping and pushing, and frequent changes in direction [2,3]. In particular, changes in direction during the game frequently involve forward, sideward, and backward movements [3]. Therefore, muscular strength and power have been postulated as the main factors that give a clear advantage in handball performance [4]. Gorostiaga et al. (2005) observed greater power levels in elite players compared to amateurs. A positive influence of strength and power on handball players’ performance level (e.g., throw velocity) was corroborated in other studies [5]. Therefore, to optimize handball players’ performance, handball training should include specific strength–power training [6,7].

In order to increase strength and power, intervention over a period between 6 and 12 weeks has been proposed [8,9]. For example, Hermassi et al. (2011) reported that elite male handball players exhibited enhanced throwing velocity, 30 m sprint performance, vertical jump, and muscle strength after 8 weeks of an in-season strength training program [8,9]. In addition, Van der Tillaar et al. (2013) found that throwing-strength training with different workloads produced improvements in throwing performance parameters, specifically, in overhead throwing with 3 kg medicine balls, which involved three series of six repetitions of throwing. This was the training load of the single-workload training group, whereas the double workload training group had to throw six series of six repetitions with the 3 kg medicine ball. The rest period between each series was around 3 min [10]. Although traditional strength training may increase force production [7], high-velocity and high rate of force development training, such as plyometric exercises, may be required [11]. In this way, plyometrics can enhance explosive contractions [12,13], and plyometric training can improve jumping performance and induce power development [14].

In particular, eccentric-overload training (EOT) using flywheel or versa-pulley inertial devices improves strength and power capacities [15,16]. The EOT induces a stretch-shortening cycle without impact [17], producing higher activation in the eccentric phase, in comparison with traditional exercises [18], and muscle activation. Although several studies have reported positive effects of EOT on strength and power gains [19], to the best of the authors’ knowledge, none of them have yet examined the effects of training programs, involving the methodologies of plyometric exercises or eccentric-overload exercises, i.e., two strength-training programs, on male handball players.

Therefore, this study mainly aimed to compare the effects of 8-week in-season strength-training programs, involving plyometric exercises or eccentric-overload exercises, on male handball players’ physical fitness, technical parameters, and physiological outcomes (i.e., jumping; sprinting; handball-specific throwing speed, and cardiorespiratory endurance).

## 2. Methods

### 2.1. Experimental Approach to the Problem

This study compared the effects of 8-week in-season training programs (plyometric vs. eccentric-overload) on male handball players’ jump, sprint, cardiorespiratory endurance, and sport-specific throwing parameters. The training program was conducted for 8 weeks (24 sessions) in season and was added to normal handball practice. The present study was conducted in ecological conditions (ecological validity), so the coaching staff and participants received input from the research team. Training data, competitive schedule, and fixture outcomes were supplied by the coaching staff of the team.

### 2.2. Participants

Handball players from a Spanish second national division club, between the ages of 18 and 22 years, were recruited. Participants were randomly assigned into three groups: control group (CG; *n* = 12; i.e., only performed handball training), plyometric training group (PG; *n* = 12), and eccentric-overload group (EG; *n* = 12). Physical performance was assessed before (pre) and after (post) the 8-week training period using a battery of tests as follows: (i) 15 m sprint time; (ii) countermovement jump; (iii) Abalakov jump; (iv) 20 m shuttle-run test; (v) throwing speed tests.

All training sessions were supervised and performed during the afternoon (before handball training). Dietary habits of the participants were maintained during the study. The players did not participate in training sessions or matches outside those scheduled for this study. All the players signed an informed consent document. All participants were adequately informed, participated voluntarily, and signed a consent form. Participants were free to withdraw from the study at any time. The inclusion criteria were the following: (a) no previous injury diagnosed within the two months before the start of the intervention nor injury diagnosed during the intervention, (b) completed 80% of the training sessions [20], (c) five or more years of experience. The participants were instructed to avoid any strenuous physical activity, other than the programmed training intervention, for the duration of the experiment. Additionally, the participants were encouraged to maintain their normal hydration levels, sleep, and dietary habits and to avoid drugs for the duration of the study. Procedures were approved by and adjusted to the Code of Ethics of the World Medical Association (Declaration of Helsinki, Fortaleza actualization, 2013) [21].

### 2.3. Testing Procedures

#### 2.3.1. General Procedure

Players were familiarized with the testing procedures a few days before the measurements. The tests were carried out for two days. The height, body mass, vertical jump, and cardiorespiratory endurance were measured on day 1. On the second day, players performed the sprinting and throwing-speed performance tests. Before testing, all players performed a standardized warm-up (7 min of running at 9 km·h^−1^ followed by 5 min of joint mobilization exercises and active stretching, and then 7 min of a specific handball warm-up (linear and curved sprints, changes in direction, jumping, passing the ball, and dribbling). The participants were also instructed to maintain their usual eating habits for the duration of the study.

Measurements were performed in the same sports hall where players trained and competed, to avoid variations in environmental or biological conditions affecting the results (humidity 42%, temperature 28.1 °C; a humidity difference of 3% and temperature difference of 13.2 °C existed between times).

#### 2.3.2. Performance Test

##### Vertical Jump Tests

The countermovement jump (CMJ) and Abalakov jump tests were performed using an electronic contact platform (Ergo Jump Plus Bosco System^®^, Muscle Lab. V7. 18, Langesund, Norway). Subjects started in a standing position with both feet together and were asked to jump as high as possible with a rapid countermovement while keeping their hands on their hips. Flight time was used to calculate the change in the height of the body’s center of gravity. The protocol for the tests has been previously reported (Ramírez-Campillo et al., 2015). An average of five trials was used for analyses. The intraclass correlation coefficient (ICC) was 0.92 for CMJ and 0.91 for the Abalakov jump.

##### 15 m Sprint Test

Sprint times (s) were recorded while athletes sprinted on an indoor synthetic surface. The sprint distance was measured with a photoelectric cell (Muscle Lab. V7.18. Ergotest Technology^®^, Langesund, Norway), with 1.2 m-high photoelectric cells. Two maximal trials were completed using 3 min of rest between trials. The ICC was 0.95.

##### Throwing Speed Tests

Players performed a 10 min standardized warm-up using a standard ball (mass = 360 g; circumference = 17.5 cm) before the throwing-speed test. Players were instructed to throw with maximal velocity toward the center of the goal from the penalty line (6 m away from the net). All throws were performed without the presence of a goalkeeper to avoid interference in the execution at maximum speed. Three types of overarm throws were made: penalty throw, a 3-step running throw, afigund a 3-step jumping throw. Each participant continued until three correct throws were recorded. Thirty seconds of rest was allowed between throws. A radar device (Stalker-Sport-Radar^®^, TX, USA) was positioned on a tripod behind the thrower to measure the throwing speed. The ICC was 0.90 for the standing throw (penalty throw), 0.88 for the 3-step running throw, and 0.89 for the jump throw.

##### 20 m Shuttle-Run Test

Participants were required to run back and forth on a 20 m pitch. They touched a 20 m line in coordination with a sound signal emitted from a pre-recorded tape. The frequency of the sound signals increased over time, involving a 0.5 km·h^−1^ increase in the running speed every minute. The test started with an initial speed of 12.52 km·h^−1^ (i.e., test stage 10). The test stopped when the subject was no longer able to follow the set pace and the total distance covered was recorded. Blood lactate concentration (basal; immediately after, and 3 min after the test) and heart rate (at the end of the test) (Polar Vantage M^®^, Kempele, Finland) were recorded. Participants did not perform strenuous activity before the test, and arrived at the laboratory 30 min in advance, during which period a basal blood sample was obtained (Blood Lactate Scout analyzer^®^, SensLab GmbH, Leipzig, Germany).

### 2.4. Training Procedures

The three groups completed handball training sessions 5 days per week on an official synthetic handball court, using appropriate handball equipment. The experimental groups performed 8 week of plyometric or EOT three days per week (M-W-F). The control group only performed regular handball training.

Each training session was performed for 60 min—warm-up (10 min), 45 min of intervention (either plyometric or eccentric-overload), and cool down (5 min). The plyometric group performed countermovement jumps with and without external load, burpees, jump lunges, alternative leg bounds, double-leg speed hops, and medicine ball throws. All plyometric sessions were conducted on a sand surface [22]. The eccentric exercises consisted of versa-pulley unilateral press and pull, versa-pulley elbow flexion and extension, YO-YO squat, and YO-YO leg-curl. The resting period between sets was 3 min. The training load was quantified each session (Foster et al., 2001). Participants received instructions and familiarization with the training exercises before the intervention. The training program performed is outlined in Table 1.

### 2.5. Statistical Analyses

Results were expressed as mean ± SD. The homogeneity of variance across groups was analyzed by Levene’s test and the Shapiro–Wilk test was used to evaluate the normality of the distribution within the data. Data were analyzed using three (PG vs. EG vs. controls) × two (pre–post) factorial ANOVA with Bonferroni post hoc comparisons. Cohen’s d effect size (ES) was calculated and interpreted as small (*d* = 0.20–0.49), medium (*d* = 0.50–0.79), or large (*d* > 0.80). Data analyses were conducted using PASW Statistics 21 (SPSS^®^, Inc., Chicago, IL, USA). The alpha level was set at *α* ≤ 0.05.

## 3. Results

At baseline, no significant differences among groups were observed in any of the descriptive characteristics (Table 2) or dependent variables (Table 3). The sRPE was similar for PG (361 ± 12.2; 95% confidence interval, 356–366) and EG (370 ± 13.3 AU; 95% confidence interval 362–377). Experimental groups similarly improved CMJ, Abalakov jump, penalty throw, 3-step running throw, and jump throw (all *p* < 0.05; Δ = 5.0–9.2%; small to large ES [0.45–1.96]) when compared to the control group. However, interventions did not affect 15 m linear sprint time, cardiorespiratory endurance, nor heart rate or blood lactate concentrations after the endurance test.

## 4. Discussion

This study aimed to compare the effects of two 8-week in-season strength-training programs on handball players’ physical and technical parameters, with 36 male handball players randomly separated into three different groups: a control group, a plyometric training group, and an eccentric-overload training group. Our novel results confirm that 8 weeks of plyometric or EOT intervention similarly improves jumping and handball-specific throwing-speed performance in male handball players. On the contrary, interventions did not affect 15 m, cardiorespiratory endurance, nor heart rate or blood lactate concentration after the endurance test.

The benefits of plyometric training on hard [23] and sand surfaces [22] have been studied previously. Our new results with competitive male handballers confirm that plyometric training on sand can improve participants’ physical fitness and sport-specific parameters (ES = 0.73–1.07; large), as can eccentric-overload training (ES = 0.45–0.53; medium). These results are in consonance with other studies, which showed that practice with jumps in dry sand produced a significant increase in power performance in the lower limbs regarding harder surfaces or grass [22,24]. In this sense, Impellizeri et al. (2008), compared plyometric training in grass and on dry sand, finding a significant improvement in squat jumps in the group that trained on dry sand [24], in addition to reducing the joint impact to which handball players were subjected. However, a novelty of this study was the eccentric-overload training approach in the EG, which showed similar gains compared to the PG. The power improvement achieved in EG may be attributed to the fact that eccentric-overload exercises increase muscle activity, improving mechanical power output and maximal strength performance [18]. In addition, contrary to traditional strength training, devices employed by the EG have been designed to provide a maximal resistance load during the full concentric phase [25], which could explain the significant improvements in jump performance observed in the EG. Therefore, future studies could analyze this intervention with handball players.

In contrast, the 15 m linear sprint was not improved in the PG or EG (ES = 0.25–0.33; small). The lack of sprint time improvement may be because the participants did not perform specific sprint exercises [26]. Another potential reason could be that experimental groups included mostly exercises with a vertical force predominance, while linear sprint speed requires both vertical and horizontal, particularly the latter for short sprint distances (in this case, 15 m), given that a training program with more horizontal displacement would improve the acceleration [23,26,27]. However, this potential hypothesis must be contrasted with handball players. A third possible hypothesis for the lack of 15 m linear sprint improvement might be the high number of sprints, accelerations, and decelerations that athletes routinely perform during habitual handball practices [6,7,28].

Although both the PG (ES = 0.51; medium) and EG (ES = 0.21; small) achieved medium and small improvements in endurance performance, respectively, these were not significantly different compared to the control group. Although cardiorespiratory endurance may improve with a combined sport-specific and strength and conditioning approach [29], previous studies also noted a lack of endurance enhancement after strength training [30]. These findings may be particularly common in highly trained athletes with highly developed endurance capabilities, such as handball players, though there are no previous studies to the best of the authors’ knowledge. Alternatively, a lack of training specificity may also explain the lack of endurance improvement. Some exercises such as jump-rope may have provided greater endurance stimulation [31]. Thus, future studies should analyze if greater volume and density of treatment might have favored improvements.

The throwing-speed performance is highly dependent on throwing technique, upper- and lower-body and trunk strength, and vertical jumping ability [4,32]. Moreover, resistance training with high load (60–80% 1 RM) influenced throwing speed performance positively [33]. In our study, the PG and EG improved all throws in which the lower limbs were involved in the specific throwing action (penalty throw, 3-step running throw, and jump throw), confirming previous findings in handball players—although at different competitive levels [4] and ages [32]—that reported significant correlations between the strength of the lower limbs and throw speed [4,32]. Therefore, strength improvement in the lower limbs could improve the speed of specific throws in handball, similar to other sports [33].

Future studies may analyze resistance-type circuit training programs in order to improve the speed of specific throws for handballers.

## 5. Conclusions

In summary, an 8-week handball intervention, performing plyometric or eccentric-overload training in-season, improves the physical and technical parameters of highly trained male players when compared to regular handball practice.

### 5.1. Limitations, Strengths and Future Research

It should be noted that it is difficult to obtain larger sample sizes of athletes, as not many have the availability to comply with the training and supplementation instructions required by the study. Moreover, sampling using a convenient, non-probabilistic sampling procedure may produce results that are not representative of the rest of the population. This study was probably not blinded, in that the players performing the normal training probably knew that the other players were doing something far different in addition to normal training. Another limitation could be that it could be argued that the control group should have been a “traditional strength training” group, or that a group such as this would have been desirable if a sufficiently high number of players was available. Finally, a potential limitation is that the workloads among groups were different. These limitations may underrepresent the results and may affect study outcomes. Nevertheless, the purpose of this study was not to transfer information to the general population, but rather only to present a practical approach in this population.

However, the methodology used in this intervention was the most important strength, with ecological validity in the real sports world. Additionally, to the best of the authors’ knowledge, no previous interventions have been applied in handballers.

Future research should continue the study of the long-term effects of intervention related to neuromuscular strength, in order to expand the existing knowledge about this potential practice. It should also examine the efficacy of these types of drills in experienced athletes, to determine whether the use of these concepts as part of treatment could increase athletic performance. In addition, it should analyze how this possible practice affects the female population in handball and team sports, given that this study only focused on men to measure their performance.

### 5.2. Practical Applications

Handball players can enhance jumping, change in direction, and throwing performance by undertaking an 8-week plyometric (CMJ, burpee, jump lunge, leg bounds) or EOT (VP-unilateral press, VP-unilateral pull, VP-push press, YO-YO squat) in-season program. The performance improvements shown in this study are of great interest for handball coaches and are directly applicable to handball players because the performance of this sport relies greatly on the specific on-field vertical jump, maximal sprint, and agility abilities that were enhanced by the high-intensity-oriented training regimen. Moreover, for young handball players who do not have previous experience with plyometric and EOT training, a general adaptation phase should be scheduled to ensure proper movement technique and safety.

## Figures and Tables

**Table 1:** Plyometric training and eccentric training programs description.

Weeks	1–2	3–4	5–6	7	8
Sessions	1–6	7–12	13–18	19–21	22–24
Plyometric training					
Countermovement jump	3 × 10 *	3 × 12	3 × 15	3 × 18	3 × 20
Countermovement jump loaded (kg)	3 × 5 (10)	3 × 8 (10)	3 × 10 (12)	3 × 12 (12)	3 × 15 (15)
Burpee	3 × 5	3 × 8	3 × 10	3 × 12	3 × 15
Jump lunge	3 × 5	3 × 8	3 × 10	3 × 12	3 × 15
Alternative leg bounds	3 × 10	3 × 12	3 × 15	3 × 18	3 × 20
Double leg speed hops	3 × 10	3 × 12	3 × 15	3 × 18	3 × 20
Medicine ball throw (kg)	3 × 10 (1)	3 × 10 (1)	3 × 10 (1)	3 × 10 (2)	3 × 10 (2)
Eccentric-overload training					
VP-unilateral press	3 × 5	3 × 6	3 × 7	3 × 8	3 × 10
VP-unilateral pull	3 × 5	3 × 6	3 × 7	3 × 8	3 × 10
VP-push press	3 × 5	3 × 6	3 × 7	3 × 8	3 × 10
VP-elbow flexion	3 × 5	3 × 6	3 × 7	3 × 8	3 × 10
VP-elbow extension	3 × 5	3 × 6	3 × 7	3 × 8	3 × 10
YO-YO squat (kg)	3 × 10 (1)	3 × 10 (1)	3 × 10 (1)	3 × 10 (2)	3 × 10 (2)
YO-YO leg-curl (kg)	3 × 10 (5)	3 × 10 (5)	3 × 10 (5)	3 × 10 (7)	3 × 10 (7)

VP: Versa-pulley eccentric device; YO-YO: eccentric device. A rest period between sets of 90 s was used for all exercises; *: 3 × 10 denotes three sets of 10 repetitions each.

**Table 2:** Participants’ descriptive characteristics.

	Age (Years)	*p* Value	Height (cm)	*p* Value	Body Mass (kg)	*p* Value	Handball Experience (Years)	*p* Value	Matches/Year	*p* Value
Plyometric group (*n* = 12)	19.8 ± 2.2	0.331	178.3 ± 4.3	0.128	79.1 ± 8.3	0.236	6.2 ± 2.8	0.425	25.3 ± 7.7	0.376
Eccentric-overload group (*n* = 12)	20.5 ± 2.5	0.331	179.7 ± 3.7	0.128	81.2 ± 5.2	0.236	6.5 ± 2.8	0.425	26.3 ± 6.1	0.376
Control group (*n* = 12)	20.6 ± 1.6	0.331	180.2 ± 2.8	0.128	81.2 ± 5.2	0.236	6.3 ± 2.8	0.425	25.2 ± 5.8	0.376

**Table 3:** Changes in dependent variables before and after intervention.

	Before	After	Δ%	ES
CMJ (cm)				
Plyometric training	40.34 ± 3.9	43.22 ± 2.7 *	7.13	0.73
Eccentric training	38.22 ± 4.2	40.15 ± 3.6 *	5.04	0.45
Control	37.54 ± 4.9	38.15 ± 4.8 &	1.62	0.12
Abalakov jump (cm)				
Plyometric training	43.18 ± 3.7	47.14 ± 2.9 *	9.17	1.07
Eccentric training	41.96 ± 4.8	44.55 ± 4.4 *	6.17	0.53
Control	42.72 ± 5.1	43.87 ± 5.3 &	2.69	0.22
15 m sprint (s)				
Plyometric training	2.36 ± 0.18	2.30 ± 0.45	2.54	0.33
Eccentric training	2.34 ± 0.08	2.32 ± 0.12	0.85	0.25
Control	2.46 ± 0.11	2.43 ± 0.09	1.21	0.27
20 m SRT (s)				
Plyometric training	322 ± 17.45	331 ± 19.49	2.79	0.51
Eccentric training	328 ± 20.67	334 ± 18.34	1.82	0.29
Control	327 ± 20.18	331± 21.19	1.22	0.19
20 m SRT HRmax (bpm)				
Plyometric training	192.52 ± 6.9	193.34 ± 5.6	0.42	0.11
Eccentric training	190.12 ± 10.2	192.26 ± 10.2	1.12	0.20
Control	189.25 ± 9.4	190.83 ± 7.3	0.83	0.16
20 m SRT lactate (mmol/L) ^¥^				
Plyometric training	6.55 ± 1.67	6.68 ± 1.77	1.98	0.07
Eccentric training	6.48 ± 2.18	6.44 ± 2.61	0.62	0.01
Control	6.73 ± 1.51	6.78 ± 1.64	0.74	0.03
Penalty throwing velocity (km/h)				
Plyometric training	83.77 ± 3.54	90.41 ± 4.88 *	7.92	1.87
Eccentric training	81.32 ± 2.78	86.01 ± 2.46 *	5.76	1.68
Control	81.93 ± 3.66	82.41 ± 4.27 &	0.58	0.13
3-step running throw velocity (km/h)				
Plyometric training	87.56 ± 5.29	93.61 ± 5.12 *	6.90	1.14
Eccentric training	84.15 ± 4.11	90.76 ± 4.34 *	7.85	1.60
Control	83.75 ± 5.29	84.68 ± 5.94 &	1.11	0.17
Jump throw velocity (km/h)				
Plyometric training	82.10 ± 3.62	89.21 ± 3.89 *	8.66	1.96
Eccentric training	80.62 ± 4.16	85.14 ± 4.25 *	5.60	1.08
Control	80.00 ± 3.22	81.23 ± 3.58 &	1.53	0.38

Legend: &: significant difference compared to the control group (*p* < 0.05); *: significant difference between value sbefore and after intervention (*p* < 0.05); ^¥^: 3 min after the 20 m SRT; HR_max_: maximal heart rate; SRT: shuttle-run test for cardiorespiratory endurance.

## Data Availability

Not applicable.
